# Integrated behavioral health care in pediatric practices: the dollars don’t add up

**DOI:** 10.1093/haschl/qxaf046

**Published:** 2025-03-06

**Authors:** Jane M Zhu, Sandy Chung, Mary Giliberti

**Affiliations:** Division of General Internal Medicine, Oregon Health & Science University, Portland, OR 97239, United States; Trusted Doctors, Fairfax, VA 22033, United States; Mental Health America, Alexandria, VA 22314, United States

**Keywords:** behavioral health, youth, integrated care, pediatrics, practice management

## Abstract

Given the prevalence of behavioral health disorders in children and adolescents, and ongoing access gaps, clinicians and policymakers have pushed to expand integrated care models in pediatric primary care settings. Despite the evidence surrounding the efficacy of integrated behavioral health models for pediatric populations, uptake has been slow. Practices report many implementation barriers, including stand-up costs, training needs, and inadequate administrative support. In this Commentary, we argue that, perhaps even more fundamentally, ongoing financial challenges are restricting model adoption, scale, and sustainability, particularly for independent and smaller pediatric group practices. Two real-world case studies illustrate several key financial challenges and opportunity costs for such practices, including administrative barriers and lag times in contracting and credentialing behavioral health providers, reimbursement rates that fail to cover the costs of care delivery, opportunity costs for practice revenue, and persistent coding and billing restrictions. Policies aiming to fulfill the clinical promise of integrated behavioral health care must account for these fiscal realities, prioritizing billing and payment alignment with pediatric practices’ bottom dollar.

Behavioral health disorders are among the most common health concerns in children and adolescents, with a prevalence of approximately 1 in 7 youths ages 3 to 17 years.^1^ As demand for services has continued to outpace access, half of youth report unmet behavioral health needs.^1^ Evidence consistently highlights the promise of integrated care models^2^—mental health delivery centered in pediatric primary care settings—to improve access to care, treatment engagement, and provider satisfaction, particularly for those who face barriers in accessing specialty mental health services. Integrated care models allow for early identification and response to behavioral health concerns through assessments, short-term interventions, and referrals to more intensive supports through various models. These models range from care communication across different settings to dedicated interdisciplinary treatment and care-management teams co-located at the same practice.^3^ A key advantage is the ability to reach wide swaths of the pediatric population, particularly those from low-income and racial and ethnic minoritized groups.^4^

Despite the evidence surrounding the efficacy of integrated behavioral health models for pediatric populations, uptake has been slow. Practices report many implementation barriers, including initial costs, training needs, and inadequate administrative support.^5^ Perhaps most practically, the experiences of pediatric clinics suggest ongoing financial challenges that restrict model adoption, scale, and sustainability.^6^ Limited evidence, centered around adult primary care practices, demonstrates variable financial viability depending on the approach towards integration. For example, 1 study of Medicare-serving clinics simulated net revenues under a telephone-based care-management model but net losses for in-person behavioral health clinicians.^7^ However, less evidence exists within pediatric care, where integrated behavioral health models remain less common than in adult settings.

## Billing and coding restrictions with integrated care models

Various billing mechanisms supporting integrated care models present distinct financial, billing, and administrative barriers. Practices with embedded behavioral health providers must credential, and often must separately contract, those providers with insurance companies due to different payer entities overseeing behavioral health and physical health credentialing functions. Negotiated reimbursements are often influenced by the number of providers participating in the payer contract. A pediatric practice employing just 1 or 2 behavioral health providers, for instance, may not be able to leverage higher negotiated rates to cover costs of salaries, infrastructure, and overhead that may be needed to support their activities within a general practice. Furthermore, payers vary in their coverage of care coordination and navigation activities, which often constitute a large portion of time and effort for integrated behavioral health providers.

Table 1 highlights 2 real-world case studies, illustrating several key financial challenges and opportunity costs. Practice 1 uses the Collaborative Care model (assessment, brief psychological interventions, measurement-based care, and medication management by a primary care doctor assisted by a consulting psychiatrist).^8^ Collaborative care codes (eg, CPT 99492, 99493, 99494, and G2214) are billed under the primary pediatrician without the need to credential a care manager. However, providers cannot bill these behavioral health services to the practice's full set of contracted payers, because some payers will not reimburse for a behavioral health provider that operates outside of a behavioral health Independent Practice Association (IPA) specialty network. These restrictions effectively prevent some patients from accessing collaborative care services, and bars the practice from utilizing its care manager and psychotherapy staff at full capacity. Moreover, while collaborative care services use add-on billing codes, there are monthly limits on billable time. Finally, many patients have variable cost-sharing responsibilities for collaborative care services, constraining broader participation in these services within the practice.

**Table 1.** Case studies of financial challenges faced by integrated pediatric practices.

	Practice type	Rurality	Clinicians	Chief challenges	Net revenue or losses
Practice 1	General pediatrics practice; 50% Medicaid and 50% commercially insured patients	Rural	Three full-time pediatricians, 1 part-time developmental and behavioral pediatrician, 1 part-time child and adolescent psychiatrist; 2 part-time Licensed Clinical Social Workers (LCSWs); 5 part-time Licensed Master Social Workers	Payer restrictions regarding who can bill for collaborative care; limits on billable time; cost-sharing limits patient participation; reimbursement too low to cover costs of behavioral health clinician employment	−$20 000 annually
Practice 2	General pediatrics practice; 30% Medicaid and 70% commercially insured patients	Suburban	Three pediatricians and 2 nurse practitioners; 1 full-time LSCW to provide psychotherapy, counseling, and care-management services	Opportunity costs of revenue that would otherwise be generated from acute medical visits; reimbursement too low to cover costs of employment; extended delays for contracting and credentialing	−$10 000 annually to staff clinician

In fiscal year (FY) 2023, revenue for this practice from all behavioral health claims, including 1100 Evaluation and Management (E&M) visits of varying complexity and collaborative care codes, was $134 838. In comparison, costs for employing a staff of behavioral health providers, excluding pediatricians, totaled $154 698.91, amounting to a $20 000 annual loss.

The second practice uses a Primary Care–Mental Health Integration Model, offering assessment and on-site therapy through a Licensed Clinical Social Worker (LCSW) and medication management from a pediatrician with consultation from the state's psychiatric consultation services.^8^ Each pediatrician in the practice sees, on average, 3 patients daily for mental health concerns, with 575 total such visits in FY 2023. Due to reimbursement structures, mental health visits (lasting 45 minutes–1 hour) generate less revenue than multiple acute physical health appointments averaging 10–15 minutes. Specifically, the state Medicaid program pays $61.67 for a 10–15-minute physical medical appointment but only $122.17 for the longer mental health appointment, amounting to a $373.53 loss per clinician per day (assuming 3 mental health visits per clinician per day).

The presence of an integrated behavioral health provider can improve access to therapy as well as productivity of the primary clinician, provided that the practice already addresses mental health concerns in-house. In such scenarios, practices have the potential to shift complex mental health patients to an integrated behavioral health provider and allow physicians to focus on medical visits. Limited cost-effectiveness studies focused on adult primary care settings also suggest cost-savings for patients, but have not evaluated financial effects on practices.^9^ However, for practices that historically have referred to outside specialists for behavioral health services, transitions to integrated mental health visits represent an initially steep opportunity cost—for this second practice, amounting to an annualized loss of over $94 000 just for 1 clinician. Reimbursement for the practice's LCSW's sessions currently do not cover the LCSW's salary and overhead, with the practice reporting an average loss of $10 000 per year to staff a therapist.

Finally, the practice had to negotiate distinct behavioral health insurance contracts from its medical insurance contracts. Each behavioral health contract required several months to finalize, with an additional 3–6-month waiting period to credential the therapist with each insurer during which the practice was unable to bill for services.

## Lessons for policy

Policy discussions around scaling adoption of implementing integrated behavioral health care have not sufficiently addressed financial pressures faced by practices, particularly with initial model adoption. Pediatric practices without access to grant or other support mechanisms face steep opportunity costs, with financial incentives often failing to align with clinical best practices. The financial burdens facing pediatric practices in these case studies include administrative barriers and lag times in contracting and credentialing behavioral health providers, reimbursement rates that fail to cover the costs of care delivery, opportunity costs for practice revenue, and persistent coding and billing restrictions.

Alongside more empirical evidence, policies to address these barriers are needed. For instance, the federal government could more comprehensively gather information on rates paid to primary care practices, including pediatric practices, under different approaches to integrated care. The bipartisan Complete Act is currently pending to increase payment rates for integrated care models in Medicare and, if successful, could influence rates paid by private plans and Medicaid. States can convene payers to streamline credentialing processes and reduce payment delays, ensuring that managed-care contracts are clear about billing and credentialing requirements, and to allow provider organizations to make informed decisions when hiring specialty behavioral health staff.

States can also accelerate financial viability of integrated care models, in part by expanding coverage and payment for behavioral health services. For example, New Mexico enacted the No Behavioral Health Cost Sharing Act in 2021, requiring private insurers to waive copays, coinsurance, and deductibles for all behavioral health services through January 2027. Other states have also enacted legislation to support integrated care, including requiring private plans to cover separate mental health wellness visits, mandating coverage of collaborative care codes, and waiving all patient cost-sharing for behavioral health services.

In Medicaid, where reimbursement rates for behavioral health services are consistently lower than in other insurance markets,^10^ Congress should provide additional Medicaid match for integrated care codes, screening, therapy, and care management for behavioral health care providers in primary care practices. Reimbursement for collaborative care and integrated behavioral health care codes should be expanded, as only half of states currently reimburse for these services. States should also ensure that rates paid to primary care providers are financially viable for practices, using 1115 demonstrations to reimburse for higher tiers of integration, as Massachusetts recently demonstrated.

## Conclusion

Integrated behavioral health models constitute an important access point for children and adolescents to obtain behavioral health care amidst a national youth mental health crisis. Despite its promise, however, adoption and scaling of integrated behavioral health in pediatrics continues to lag expectations. Funding is needed to support more comprehensive transitions to this model, with policy efforts prioritizing billing and payment alignment with pediatric practices’ bottom dollar.

## Supplementary Material

qxaf046_Supplementary_Data
